# FIGO good practice recommendations on the use of pessary for reducing the frequency and improving outcomes of preterm birth

**DOI:** 10.1002/ijgo.13837

**Published:** 2021-09-14

**Authors:** William A. Grobman, Jane Norman, Bo Jacobsson, the FIGO Working Group for Preterm Birth, Joe Leigh Simpson, Ana Bianchi, Stephen Munjanja, Catalina María Valencia González, Ben W. Mol, Andrew Shennan

**Affiliations:** ^1^ Department of Obstetrics and Gynecology Feinberg School of Medicine Northwestern University Chicago Illinois USA; ^2^ Health Science Faculty Office University of Bristol Bristol UK; ^3^ Department of Women and Children’s Health King’s College London UK; ^4^ Department of Obstetrics and Gynecology Institute of Clinical Science Sahlgrenska Academy University of Gothenburg Gothenburg Sweden; ^5^ Department of Obstetrics and Gynecology Sahlgrenska University Hospital Gothenburg Sweden; ^6^ Department of Genetics and Bioinformatics Domain of Health Data and Digitalization Institute of Public Health Oslo Norway

**Keywords:** antenatal, child outcome, pessary, singleton, twin

## Abstract

A pessary is a device made of synthetic material that is placed in the vagina and has been used for prevention of preterm birth. It has been suggested that a potential mechanism of the pessary is alteration of the cervico‐uterine angle to a more posterior position, which reduces cervical compression in women with a singleton pregnancy and a short cervical length. Pessaries should not be used in routine clinical care to reduce the frequency of preterm birth or to improve outcomes (e.g. neonatal outcomes) related to preterm birth. In women with a twin pregnancy—regardless of cervical length—pessaries should not be used in routine clinical care to reduce the frequency of preterm birth or to improve outcomes (e.g. neonatal outcomes) related to preterm birth. Presently there is no sufficient evidence suggesting that pessaries should be used as a standard treatment to prevent preterm birth; their use should be reserved for study populations.

## Synopsis

Pessary for prevention of preterm delivery is presently not recommended and should only be used for study populations.

## 1 INTRODUCTION

A pessary is a device made of synthetic material that is placed in the vagina. One potential application of the pessary has been preventing preterm birth in high‐risk groups, such as women with a singleton pregnancy with a shortened cervix in the mid‐gestation, or those with a twin gestation. One hypothesis is that the pessary alters the cervico‐uterine angle to a more posterior position, which reduces cervical compression and other changes. Nevertheless, the exact physiologic mechanism by which a more posterior cervix would lead to a lower preterm birth rate has not been demonstrated.

There have been several randomized trials within the last decade (and many more ongoing) that have evaluated whether pessary is a beneficial strategy for preterm birth prevention in a variety of different populations (e.g. women with a singleton pregnancy and short cervix, twins and a short cervix, or twins regardless of cervical length). These trials have yielded inconsistent results even among women with similar risk factors for preterm birth. Some showed benefit among those who received a pessary, and others showed statistically similar results regardless of whether a pessary was used.^1^, ^2^

## 2 CLINICAL SCENARIOS

### 2.1 Women with a singleton pregnancy and short cervical length

As two examples of conflicting studies among women with singleton pregnancies and a short cervix, Goya et al. randomized those with a singleton pregnancy and a cervix ≤25 mm to an Arabin pessary vs no pessary, and found that those who received the pessary had an 82% reduction in the relative risk of spontaneous preterm birth and an 86% reduction in a composite of perinatal morbidity.^1^ In contrast, Nicolaides et al.^2^ used a similar study design (although added progesterone if the cervix was ≤15 mm), and found no difference in either outcome.^2^ Other investigations have produced similarly inconsistent findings.^3^, ^4^

*Recommendation*: *In women with a singleton pregnancy and a short cervical length, a pessary should not be used in routine clinical care to reduce the frequency of preterm birth or to improve outcomes (e*.*g*. *neonatal outcomes) related to preterm birth*.

### 2.2 Women with a twin pregnancy

Among a general population of women with twins, Liem et al.^5^ found no difference in gestation length between women randomized to receive a pessary or no pessary. In the study by Liem et al., the population was further stratified by several subgroups of cervical length, and in the subset with a cervical length <38 mm, those with a pessary had a significantly longer gestation and better perinatal outcomes. Dang et al. used this information to design a trial in which those with twins and a cervical length <38 mm were randomized to pessary or vaginal progesterone. There was no significant difference in the frequency of preterm birth <34 weeks (16% vs 22%; RR 0.73; 95% CI 0.46–1.18), which was the primary outcome in that study. The authors did find that some secondary outcomes (e.g. composite perinatal adverse outcomes) were significantly less frequent (albeit without adjustment for multiple comparisons) among women who received the pessary.^6^ Goya et al.^7^ showed a considerably lower chance of spontaneous preterm birth <34 weeks (RR 0.41; 95% CI 0.22–0.76) among those with twins and a cervical length ≤25 mm who were randomized to pessary, while Nicolaides et al.—who randomized women with twins regardless of cervical length—did not find any effect, even in women with a short cervical length.^7^, ^8^ Norman et al.^9^ randomly assigned 503 women with a twin pregnancy and cervical length ≤35 mm to pessary in addition to standard care or standard care alone. There was no difference in the primary obstetric outcome of spontaneous preterm birth before 34^+0^ weeks (adjusted odds ratio 0.87; 95% CI 0.55–1.38). Other investigators similarly have shown no difference in preterm birth rates among women with twins who received a pessary. However, some of these trials were smaller, with a corresponding greater chance of type II error.^10^ A meta‐analysis performed by Norman et al.,^9^ which included their own and other published data, showed that the use of cervical pessary did not result in a statistically significant reduction in preterm birth before 34 weeks in women with a short cervix (OR 0.74; 95% CI 0.50–1.11).

*Recommendation*: *In women with a twin pregnancy—regardless of cervical length—pessaries should not be used in routine clinical care to reduce the frequency of preterm birth or to improve outcomes (e*.*g*. *neonatal outcomes) related to preterm birth*.

## 3 CONCLUSION

While some studies have shown benefits from pessary, those benefits have often not been related to the a priori primary outcome or have been seen only after subgroup analysis in women with different cervical lengths. Other studies have shown statistically similar effects among women at risk of preterm birth regardless of whether they received a pessary. In some cases, the size of the trial has been small enough, and the confidence interval around the point estimate of the effect size sufficiently wide, that a clinically significant benefit remains possible. Interpretation of the results is further complicated because studies have varied concerning management among those enrolled, including whether or not vaginal progesterone was used. This inconsistency in findings and lack of clear delineation of a specific group of individuals among whom pessary is efficacious is the basis upon which to conclude that, at this time, there is not sufficient evidence to suggest that pessary should be used as a standard treatment to prevent preterm birth, and that its use should be reserved for study populations.

## CONFLICTS OF INTEREST

William A. Grobman reports no conflicts of interest. Jane Norman reports receipt of grants from government and charitable bodies for research into understanding the mechanism of term and preterm labour and understanding treatments; participation in a Data Safety and Monitoring Board for a study involving a preterm birth therapeutic agent for GlaxoSmithKline; and consultancy for Dilafor on drugs to alter labour progress. Bo Jacobbson reports research grants from Swedish Research Council, Norwegian Research Council, March of Dimes, Burroughs Wellcome Fund and the US National Institute of Health; clinical diagnostic trials on NIPT with Ariosa (completed), Natera (ongoing), Vanadis (completed) and Hologic (ongoing) with expendidures reimbused per patient; clinical probiotic studies with product provided by FukoPharma (ongoing, no funding) and BioGaia (ongoing; also provided a research grant for the specific study); collaboration in IMPACT study where Roche, Perkin Elmer and Thermo Fisher provided reagents to PLGF analyses; coordination of scientific conferences and meetings with commercial partners as such as NNFM 2015, ESPBC 2016 and a Nordic educational meeting about NIPT and preeclampsia screening. Bo Jacobbson is also Chair of the FIGO Working Group for Preterm Birth and the European Association of Perinatal Medicine's special interest group of preterm delivery; steering group member of Genomic Medicine Sweden; chairs the Genomic Medicine Sweden complex diseases group; and is Swedish representative in the Nordic Society of Precision Medicine.

## AUTHOR CONTRIBUTIONS

All authors and the FIGO Working Group for Preterm Birth drafted the concept and idea of the paper. WAG wrote the first version of the manuscript. JN and BJ revised various versions of the manuscript. All authors and working group members commented on the manuscript and approved the final version.

## MEMBERS OF THE FIGO WORKING GROUP FOR PRETERM BIRTH, 2018–2021

Bo Jacobsson (Chair), Joe Leigh Simpson, Jane Norman, William A. Grobman, Ana Bianchi, Stephen Munjanja, Catalina María Valencia González, Ben W. Mol, Andrew Shennan.
